# Genetic Associations in Acquired Immune-Mediated Bone Marrow Failure Syndromes: Insights in Aplastic Anemia and Chronic Idiopathic Neutropenia

**DOI:** 10.1155/2012/123789

**Published:** 2012-08-26

**Authors:** Irene Mavroudi, Helen A. Papadaki

**Affiliations:** Department of Hematology, University of Crete School of Medicine, P.O. Box 1352, 71110 Heraklion, Crete, Greece

## Abstract

Increasing interest on the field of autoimmune diseases has unveiled a plethora of genetic factors that predispose to these diseases. However, in immune-mediated bone marrow failure syndromes, such as acquired aplastic anemia and chronic idiopathic neutropenia, in which the pathophysiology results from a myelosuppressive bone marrow microenvironment mainly due to the presence of activated T lymphocytes, leading to the accelerated apoptotic death of the hematopoietic stem and progenitor cells, such genetic associations have been very limited. Various alleles and haplotypes of human leucocyte antigen (HLA) molecules have been implicated in the predisposition of developing the above diseases, as well as polymorphisms of inhibitory cytokines such as interferon-**γ**, tumor necrosis factor-**α**, and transforming growth factor-**β**1 along with polymorphisms on molecules of the immune system including the T-bet transcription factor and signal transducers and activators of transcription. In some cases, specific polymorphisms have been implicated in the outcome of treatment on those patients.

## 1. Introduction

Autoimmune diseases have been in the spotlight over the years and many of them seem to share similar underlying pathophysiology and immunogenetic mechanisms resulting from the interaction of multiple genetic and environmental factors [1–5]. Numerous genome-wide association studies have proven to be a useful tool in revealing the involvement of chromosomal loci that are associated with susceptibility to specific disorders [6, 7]. However, in the distinct but nonetheless related group of immunomediated bone marrow (BM) failure syndromes, there is an underexplored field of genetic associations. In this paper we will highlight such associations focusing on two diseases which share similar immunopathologic features, namely acquired aplastic anemia (AA) and chronic idiopathic neutropenia (CIN), both belonging to the group of BM failure syndromes.

## 2. Aplastic Anemia

### 2.1. Pathogenetic Features

Acquired AA is a disease characterized by a hypoplastic or aplastic BM and peripheral pancytopenia of a varying degree [8]. Although in some AA patients viral infection, drug, or chemical exposure can be linked to the disease pathogenesis, in most cases the underlying etiology remains elusive. However, numerous studies have unveiled the role of T lymphocytes in the pathogenesis of AA. Specifically, it has been shown that oligoclonally expanded self-reactive T cells [9–11] induce apoptosis of hematopoietic stem/progenitor cells [12]; this can be mediated either through an interaction via the Fas/Fas-ligand (FasL) pathway [13, 14] or by the production of proinflammatory and growth inhibitory cytokines such as tumor necrosis factor-*α* (TNF-*α*) and interferon-*γ* (IFN-*γ*) [15–19], thus resulting in a depletion of the hematopoietic stem cell pool in the BM. Other immune cells and molecules have been also implicated in the pathogenesis of acquired AA and have been reviewed elsewhere [20, 21]. The guidelines of treatment of AA suggest either hematopoietic stem cell transplantation (HSCT), which can cure the disease but it is not applicable to all patients, and/or immunosuppressive treatment (IST) with antithymocyte globulin (ATG) and/or cyclosporine A (CsA) [22–25]. The responsiveness of a significant proportion of AA patients to immunosuppressive therapy gives further evidence for the underlying immune pathophysiology of the disease and classifies it in the wide spectra of autoimmune diseases [20, 26]. However, in several cases of IST-treated patients, the development of clonal disease has been the most serious complication, where the expansion of clones and clonal progression has been attributed to an immune selection and immunological escape [27, 28].

### 2.2. Genetic Factors in Aplastic Anemia

#### 2.2.1. HLA Molecules

The human leukocyte antigen (HLA) is the most polymorphic genetic system. Its genes reside on chromosome 6 and determine HLA class I molecules encoded by HLA-A, HLA-B, and HLA-C loci, as well as HLA class II molecules encoded by HLA-DRA, -DRB1, -DRB3, -DRB4, -DRB5, -DQA1, -DQB1, -DPA1, and -DPB1 loci [29]. Given the fact that oligoclonally expanded T cells are involved in the pathophysiology of acquired AA [9–11] and since the interaction between CD8^+^ or CD4^+^ and their targets is mediated by HLA class I or II peptides, respectively, it has been suspected that polymorphic loci of these genes would be implicated in the susceptibility to the disease. Indeed, over the years various groups have tested this hypothesis in distinct populations and ethnicities.

By serological and molecular typing, it has been shown in a number of studies that HLA-DR2 was the gene associated with susceptibility to AA and in particular HLA-DRB1∗15 allele in Chinese, Japanese, and Caucasians of different ethnicities [30–34] although there have been some contradicting facts [35]. Furthermore, it has been found that the HLA-DRB1∗1501 and not the HLA-DRB1∗1502 allele was the one associated with an increased risk of developing AA [31, 36, 37] despite the fact that in a group of Japanese patients the DRB1∗1502 allele showed increased frequency, attributed mainly to the older age group [38]. However, only patients bearing the HLA-DRB1∗1501 allele and interestingly the DRB1∗1501-DQA1∗0102-DQB1∗0602 haplotype had a better response to CsA treatment [31, 38].

Other HLA alleles predisposing to development of AA have also been investigated. In a case report, the HLA-DRB1∗0405 allele was a candidate gene for susceptibility to AA [39], while in another study, high-resolution genotyping of HLA-DRB1 showed that the HLA-DRB1∗04 allele coding for alanine at position 74 (HLA-DR4-Ala74) predisposed to severe AA (SAA) independently from the DRB1∗1501 risk allele [40]. Furthermore, the DRB1∗04 alleles had a worse response to CsA and a tendency to a poor prognosis. The HLA-DRB1∗07 allele has been reported overexpressed in AA patients with no difference between adults and children, placing it as a susceptible allele for AA, at least in that cohort of Iranian subjects [35]. In addition, other candidate alleles predisposing to AA and SAA in children have been reported such as HLA-B∗48:01, HLA-DRB1∗09:01, and HLA-B14 [41, 42]. However, in the latter study [42], it was demonstrated that different HLA associations occur in children and adults; therefore any assumptions regarding HLA allele distribution between these groups should be made cautiously.

Beside risk alleles, there have been studies revealing the possible protective role of HLA variants in developing AA. The allele HLA-DRB1∗13 appeared to be protective in SAA children of Turkish origin [43], as well as the HLA-DRB1∗03:01, HLA-DRB1∗11:01, and HLA-B∗51:01 alleles in Chinese children with AA [41]. Likewise, in a small cohort of Pakistani AA patients, HLA-DRB1∗03 had higher frequency in controls suggesting a putative protective role [33]. In addition, in the Korean population, the DRB1∗1302 allele has been found significantly lower in the group of SAA patients compared to controls or non-SAA patients [36]. However, in the previously mentioned study, the haplotype A∗31-B∗51-DRB1∗13 was associated with predisposition to AA along with the A∗02-B∗40-DRB1∗15 and A∗33-B∗58-DRB1∗15 haplotypes [33].

In a large-scale single-nucleotide polymorphism (SNP) array-based study concerning a Japanese AA patient cohort, copy number-neutral loss of heterozygosity (CNN-LOH) of the 6p arm (6pLOH) was detected in a substantial proportion of patients [44]. The HLA-A∗02:01, HLA-A∗02:06, HLA-A∗31:01, and HLA-B∗40:02 alleles were overrepresented in this population, and in the 6pLOH(+) clones, the missing HLA alleles were biased towards the four alleles mentioned above. This observation has led to the hypothesis that since cytotoxic T cells that presumably target antigen(s) present on hematopoietic stem cells through specific HLA class I molecules, 6pLOH(+) cell clones found in AA patients may have been derived by a progent that managed to escape the autoimmune attack by effectively deleting the risk of HLA species responsible for the immune insult. However, this escape mechanism from autoreactive cytotoxic T cells could not render 6pLOH(+) stem cells able to repopulate the BM effectively, unless immunosuppressive treatment was applied. The later observation was possibly due to the presence of inhibitory cytokines such as IFN-*γ* and TNF-*α* in the BM of AA patients [44]. Nevertheless, future studies should be designed to shed light into the origin of autoimmunity, predisposition to the disease, and outcome of treatment regarding the thousands of HLA variants and the different emerging haplotypes in distinct ethnic populations.

#### 2.2.2. Myelosuppressive Cytokines and Molecules

Elevated levels of inhibitory cytokines such as IFN-*γ* and TNF-*α* as well as elevated levels of Fas antigen on CD34^+^ progenitor cells in the BM of AA patients have been previously reported to play a key role in the pathophysiology of the disease [13, 45–47]. Polymorphisms in such cytokines have been investigated by various groups in order to expose a genetic predisposition to AA or the outcome of IST.

The TNF-*α* gene −308 promoter/enhancer polymorphism, and specifically the TNF2 allele (−308A), has been associated with elevated TNF-*α* levels [48] and has been shown to be overrepresented in a SAA Chinese population, contributing to the susceptibility to the disease in a DR3- and DR4-independent manner [49]. However, no susceptibility was demonstrated in milder forms of AA [49], which is consistent with other observations where the distribution of the TNF2 allele did not differ between AA patients and controls [50, 51]. Nevertheless, there are contradicting observations where the specific −308 AA TNF-*α* genotype was overrepresented in the AA group of patients [52]. Likewise, although in a German group the response to immunosuppressive therapy due to this rare allele was better even after 3 months of treatment compared to noncarriers [50], in another group such association was not demonstrated [51]. The different outcomes of the studies might be attributed to the low number of subjects tested and to variations due to a nonhomogenous population with AA of varying degree and different ethnicities.

The IFN-*γ* +874 A/T gene polymorphism, and in particular the +874TT genotype, has been shown to result in elevated levels of IFN-*γ* production [53]. Many groups have demonstrated that the TT genotype is overrepresented in AA patients and correlates with susceptibility to the disease but not with the disease severity [52, 54–56]. Moreover, it has been shown that the above specific genotype might predict a good response to IST [55]. Other polymorphisms such as the −2,353 A/T rs7139169 and the −1,616 C/T rs2069705 have also been studied in AA, and it has been shown that the minor T allele of the former was protective and reduced the risk for AA, as well as the haplotype TCA regarding the polymorphisms in −2,353, −1,616, and +874 of IFN*γ* gene. In addition, the above-mentioned −2,353 T allele and TCA haplotype was shown to induce resistance to IST [51]. Polymorphisms of a CA repeat microsatellite sequence in the first intron of the IFN-*γ* gene have been also shown to affect the production of IFN-*γ*. Specifically, homozygosity for the 12 (CA) repeats in position 1349 of the gene results in production of higher levels of IFN-*γ* [53, 57]. The frequency of the 12-12 (CA) genotype as well as the single allele 12 in Caucasian and Chinese AA patients has been shown to be higher than controls, thus associating this variable number of dinucleotide repeat (VNDR) 1349 of IFN-*γ* to the risk of AA [57, 58].

Transforming growth factor-*β*1 (TGF-*β*1) is another cytokine playing a role in hematopoiesis with multifunctional effects. In patients with aplastic anemia, the frequency of genotypes associated with high production of TGF-*β*1 and in particular the −509 TT genotype was shown to be increased as opposed to controls [52, 56, 59]. This is in contrast to the fact that lower levels of TGF-*β*1 have been described in the serum and in vitro cultures of AA patients [60]. However, the expression levels in the periphery do not always reflect the levels of locally expressed cytokines in the BM. Other polymorphisms of TGF-*β*1 like −590 C/T rs1800469 as well as the P10L C/T rs1800470 have been reported to play no role in the susceptibility to AA. However, the T allele of the P10L C/T, along with the CT haplotype regarding the above two polymorphisms, has demonstrated higher response to IST even at the third month of treatment compared to patients lacking this haplotype [51].

 Polymorphisms of molecules like FAS and various interleukins (IL) such as IL-1*β*, IL-2, IL-6, IL-10, and IL-12 that have a role in the pathogenesis of AA have been investigated by different groups, but no significant difference was observed between patient groups and controls [51, 52, 56, 59].

#### 2.2.3. Other Immune Molecules

Increased expression of IFN-*γ*, TNF-*α*, and IL-2 from AA patients indicates that hematopoietic stem and progenitor cells are destroyed through a T-helper (Th)1 cell response [61]. T-bet or TBX21 belongs to the T-box family of transcription factors, it is the key regulator of Th1 development and function, and it is found in Th1 but not in Th2 cells [62, 63]. In patients with AA, T-bet is found elevated and transcribes actively the IFN-*γ* gene [64]. TBX21 has been suggested as a common risk gene for a variety of autoimmune disorders [65, 66]. Interestingly, the C allele of T-1993C, the TBX21 gene promoter, was associated with decreased risk in AA [67].

 Signal transducer and activator of transcription 4 (STAT4) is a transcription factor binding to genes encoding T-bet and IFN-*γ* and plays a critical role in Th1 and Th17 cell differentiation [68]. Polymorphisms of the STAT4 gene have been associated with various autoimmune diseases [69–71]; among the polymorphisms tested, the rs7574865 was a candidate common risk polymorphism. In a cohort of Chinese population, the rs7574865 polymorphism was found to pose as a candidate susceptibility gene, with an increased frequency of the T allele and THE TT genotype [67]. However, no association between the-above mentioned polymorphism and the response to IST was established.

 Molecules that are expressed on T cells affecting self-tolerance and autoreactivity have been extensively studied in autoimmune diseases. Cytotoxic T lymphocyte antigen 4 (CTLA4) is a molecule expressed on activated T cells that downregulates T cell autoreactivity [72]. Polymorphisms that result in a lower expression of CTLA4 [73, 74] have been associated with other autoimmune diseases [75]. Nonetheless, such associations were not observed in AA patients in a cohort of an Italian population [76].

 Protein tyrosine phosphatase non-receptor-type 22 (PTPN22) gene encoding for a protein tyrosine phosphatase contributes to the modulation of negative T cell selection in the thymus and downregulation of autoreactive T cells in the periphery [77]. Although polymorphisms in this gene have been associated with autoimmune disorders [78], no contribution to the susceptibility in AA was observed, at least for the PTPN22 620W allele [79].

## 3. Chronic Idiopathic Neutropenia

### 3.1. Pathogenetic Features

Chronic idiopathic neutropenia (CIN) of adults is benign disorder of granulopoiesis representing the mild form of the spectrum of BM failure syndromes. It is an acquired form of neutropenia characterized by a prolonged and unexplained reduction in the number of circulating neutrophils below the lower limit of the normal distribution [80], although other forms of mild cytopenias might coexist [81, 82]. Similar to AA, the pathogenetic cause of neutropenia in CIN is attributed to impaired BM granulopoiesis due to an inhibitory effect of the BM microenvironment consisting of activated T lymphocytes [83, 84] and monocytes [85], proinflammatory mediators, and proapoptotic cytokines such as TNF-*α*, IL-1*β*, TGF-*β*1, IL-6 as well as IFN-*γ*, and FasL [80, 83, 84]. It has been documented that progenitor and precursor cells, especially in the CD34^+^/CD33^+^ compartment, are depleted through an apoptotic mechanism implicating the FAS/FasL as well as the CD40/CD40L pathways in the presence of TNF-*α* [86, 87]. Treatment of CIN patients with G-CSF administration is only recommended in the rare cases of patients suffering from severe or frequent infectious episodes [88].

### 3.2. Genetic Factors in CIN

#### 3.2.1. HLA Molecules

The major HLA alleles have been typed in a small cohort of a genetically homogenous population in Crete, Greece [89]. Of all the alleles tested (HLA-A, -B, -C, -DRB1, -DQB1, and -DPB1), only the HLA-DRB1∗1301 haplotype was significantly elevated in CIN patients as opposed to controls or other alleles. This was the first report of a genetic association to the predisposition of developing CIN. However, larger cohorts of patients need to be tested for a stronger association between HLA alleles and risk of CIN.

#### 3.2.2. Myelosuppressive Cytokines and Molecules

The involvement of elevated proinflammatory and myelosuppressive cytokines such as TNF-*α*, IL-1*β*, IL-6, TGF-*β*1 and FasL in the pathophysiology of CIN is well established [83, 90, 91]. A possible association between the elevated levels of the above soluble mediators and the genetic predisposition for CIN has been investigated by two different groups. Although the −308G/A polymorphism of the TNF-*α* gene, especially the TNF2 allele, had been previously shown to contribute to increased serum levels of this cytokine in other disease entities [92, 93], no association was identified between this polymorphism with either the occurrence or the severity of neutropenia in CIN subjects [94, 95]. Likewise, no difference in frequency of the −511C/T IL1B and the +3953C/T IL1B SNP or the variable number tandem repeat (VNTR) in intron 2 of IL-1Ra gene (IL1RN) was detected in CIN patients, [95] although these polymorphisms have been associated with IL1B gene expression and increased IL-1*β* production [96, 97]. In the same cohort, the −174G/C SNP that has been associated with altered gene expression [98] failed to associate with CIN [95]. In the same context, the frequency of the −844T/C SNP of the FasL gene was not associated with CIN [94].

Interestingly though, out of three SNPs on the TGF-*β*1 gene, namely, the −509C/T, +869T/C, and the +915G/C, the −509C/T and specifically the T allele and the TT genotype occurred in a statistically higher frequency in CIN patients thus associating this genotype with the risk of development of CIN. However, it did not associate with the severity of neutropenia. Nonetheless, patients with the CT or the TT genotype displayed elevated levels of TGF-*β*1 in the serum or long-term BM cultures, indicating a contributory role of this cytokine in the pathophysiology of the disease [94].

## 4. Closing Remarks

Genetic factors that predispose to various disease states, including the acquired immune-mediated BM failure syndromes, may contribute to the pathophysiology of these disease entities. However, isolated SNPs are unlikely to be the only regulators of the complex mechanisms taking place in such autoimmune or immune-mediated conditions, but it is rather the specific combination of genotypes of cytokines and molecules on immune cells that predispose to AA and CIN and sustain the myelosuppressive BM microenvironment along with environmental factors. Wide-scale studies on different ethnic populations, with homogenous disease characteristics, would facilitate the systemic research for associations of genetic factors and disease risk.
